# Transaxillary Breast Augmentation: A Randomized Controlled Trial Comparing a New Semiendoscopic Video-Assisted Technique versus the Blind Technique

**DOI:** 10.1097/PRS.0000000000012546

**Published:** 2025-10-20

**Authors:** Dominik Boliglowa, Martina Pontillo, Nunzio Velotti, Nicola Rocco

**Affiliations:** Kraków, Poland; and Naples, Italy; From 1 private practice, “Dominik Boliglowa Chirurg Plastyk”; 2 Department of Advanced Biomedical Sciences, University of Naples Federico II; 3 Breast Unit, University Hospital Federico II.

## Abstract

**Background:**

The pursuit of optimal aesthetic outcomes in breast surgery has led to the evolution of various surgical techniques. The transaxillary approach leads to high patient satisfaction due to its aesthetic advantage of avoiding visible scars on the breast. This approach is particularly useful in procedures such as breast augmentation and reconstructive surgery after breast cancer treatment, in which achieving an optimal cosmetic outcome is key. In this study, the authors compared the transaxillary blind approach with their new semiendoscopic video-assisted transaxillary approach, and analyzed the benefits and limitations of each method.

**Methods:**

A total of 118 patients were randomized to either the transaxillary blind approach or the semiendoscopic video-assisted transaxillary approach for breast augmentation from April of 2019 through December of 2022.

**Results:**

The results indicate that the semiendoscopic video-assisted approach required longer operative times but was associated with a lower incidence of complications, including asymmetry (eg, bottoming out, double-bubble deformity), breast animation deformity, and capsular contracture.

**Conclusions:**

This randomized trial revealed advantages and limitations of the transaxillary blind approach and a new semiendoscopic video-assisted transaxillary approach, highlighting that endoscopic assistance significantly improved surgical outcomes while maintaining an acceptable surgical time.

Breast augmentation is one of the most frequently performed operations in aesthetic surgery. The primary goal is to enhance the natural proportions of the breast and create a more symmetric, aesthetically pleasing profile. The transaxillary approach for breast surgery, first described by Hoehler in 1973,^1^ leads to high patient satisfaction due to its aesthetic advantage of avoiding visible scars on the breast.^2^ There are several approaches to breast surgery related to the type of implant and the surgical technique.^3^ Implants are available in various forms, shapes, and sizes. Textured implants are designed to reduce the risk of implant rotation and capsular contracture.^4^ The implant can be positioned in the subglandular plane, in the submuscular plane, or in the dual-plane approach, where the implant is partially under the muscle and partially under the breast tissue, offering the benefits of both the subglandular and submuscular approaches.^5,6^

A subfascial implant placement has been advocated by several authors^7–9^ as a compromise between subglandular and submuscular planes; however, others argue that the pectoralis fascia does not provide adequate support for the implant in the lower pole, where it is very thin.^10^

The incision can be made in different locations of the breast, such as the inframammary fold (IMF), in the periareolar area, or at the level of the axilla, and historically has been done through an umbilicus. Each technique has specific advantages depending on the patient’s anatomy, desired outcomes, and recovery considerations. A thorough consultation with a breast surgeon is essential to choose the most appropriate method.^11^

Surgery through a transaxillary approach can be performed blindly or using endoscopic video assistance. The blind technique is rapid, allowing the surgery to be performed within approximately 30 minutes, regardless of plane of the implant insertion. The video-assisted technique is more precise, but requires considerably longer operating times, often exceeding those of other approaches that allow direct visualization of the breast tissue.^7,12,13^

The aim of this study was to determine whether a new semiendoscopic, video-assisted transaxillary approach, combining the advantages of the traditional blind method with the enhanced visualization and precision of endoscopic surgery, offers superior outcomes compared with the conventional blind technique, particularly through its ability to consistently achieve a true dual-plane pocket.

## PATIENTS AND METHODS

### Study Design

A parallel-group randomized trial was conducted to compare the traditional transaxillary blind submuscular approach with a semiendoscopic approach, in which the videoendoscope was applied only at the end of the pocket preparation to create a proper dual-plane pocket and to ensure proper hemostasis. All surgical procedures were performed by the same experienced plastic surgeon (D.B.) in the Plastic and Reconstructive Surgery Unit of the Private Hospital Św. Róża in Kraków, Poland, or in Clinic Allmedica, Nowy Targ, Poland. Written informed consent was obtained from all enrolled patients before randomization at the preoperative appointment approximately 1 month before surgery.

Patients older than 18 years, who had undergone no previous breast surgical procedures, desired primary breast augmentation, and qualified for submuscular or dual-plane approaches with round smooth or nanotextured silicone implants were included. Patients with significant anatomical abnormalities of the breast, previous breast surgical procedures, or conditions that could significantly affect aesthetic outcomes were excluded (Table 1). Patients who underwent subfascial or subglandular augmentation, had oval-based implants, or did not attend the 1-year follow-up visit were also excluded. Baseline demographic characteristics were collected before surgery with a standardized form.

**Table 1.** Baseline Characteristics^a^

Characteristics	Group 1 (Blind Transaxillary Approach) (*n* = 59)	Group 2 (Endoscope-Assisted Transaxillary Approach) (*n* = 59)	*P*
Age, yrs	33 (29–37)	32 (29–36)	0.13
Body mass index	21 (20–24)	21 (20–23)	0.51
Implant size, cc	330 (380–305)	330 (360–305)	0.57
Ptosis grade 1^b^	9	20	0.01
Ptosis grade 2^c^	8	5	0.37
Asymmetric nipple position	19	18	0.84
IMF asymmetries	18	11	0.13
Chest abnormalities	3	4	0.69

a Data are presented as median (interquartile range) or *n* (%).

b Invisible IMF in relaxed position at frontal view.

c Invisible IMF in relaxed position as well as with elevated arms.

The choice of implant in both groups was based on the same criteria. The base diameter was selected to be 0.5 cm smaller than the actual breast width and did not exceed twice the distance between the existing or planned IMF and the nipple, measured over the sternum with arms elevated. The implant projection was determined by evaluating the amount of tissue in the lower pole. The distance from the implant base to its peak was 1 to 2 cm shorter than the distance between the IMF and the nipple, depending on the thickness of the overlying tissue.

All eligible patients were informed of the risks and benefits of the proposed procedures, received detailed information about the study, and provided written informed consent to participate. The procedures performed in the study were in concordance with the ethical standards of the institutional or national research committee and the 1964 Declaration of Helsinki and its subsequent amendments or comparable ethical standards.

### Randomization

Patients were randomized immediately before surgery by the surgeon using a remote-site 24-hour digital system that randomized patients using a computer-generated sequence. The randomization was carried out without any restriction. The assigned intervention was communicated immediately to the operating surgeon by the assistant.

### Surgical Technique

With the patient placing her hands on her hips, a 2- to 3-cm line is drawn from the deepest point of the axillary hollow, extending posteriorly along either the superior or inferior axillary crease. From this same starting point, a shorter 0.5- to 1-cm line is drawn at a right angle to the first, running parallel to the lateral border of the pectoralis major. The resulting incision measures approximately 2.5 to 3 cm, which is sufficient to accommodate the endoscopic hook; it can be extended to 4 to 4.5 cm when particularly large implants are used.

Under general anesthesia, the patient is positioned supine with her arms extended at a 90-degree angle to her body. The incision lines are then infiltrated with 15 mL of Klein solution. A 2- to 3-cm skin incision is made using a no. 15 blade, penetrating only the skin. Some authors have described a Z-shaped incision to facilitate dissection of the pectoralis muscle.^14^ Maintaining a strictly subcutaneous plane during dissection is essential to avoid critical structures in the axillary region, such as lymph nodes, nerves, and major blood vessels. (**See Video 1 [online]**, which demonstrates a 2- to 3-cm skin incision being made using a no. 15 blade, penetrating only the skin. Metzenbaum scissors are then used to separate the skin from the subcutaneous tissue in both caudal and medial directions until reaching the pectoralis major muscle. Maintaining a strictly subcutaneous plane during dissection is essential to avoid critical structures in the axillary region, such as lymph nodes, nerves [the thoracodorsal, long thoracic, and intercostobrachial nerves], and major blood vessels [the lateral thoracic vessels, the axillary vein, and the thoracodorsal artery and vein].)

**Video 1.** This video demonstrates a 2- to 3-cm skin incision being made using a no. 15 blade, penetrating only the skin. Metzenbaum scissors are then used to separate the skin from the subcutaneous tissue in both caudal and medial directions until reaching the pectoralis major muscle. Maintaining a strictly subcutaneous plane during dissection is essential to avoid critical structures in the axillary region, such as lymph nodes, nerves (the thoracodorsal, long thoracic, and intercostobrachial nerves), and major blood vessels (the lateral thoracic vessels, the axillary vein, and the thoracodorsal artery and vein).

Blunt dissection is performed under the lateral border of the pectoralis major muscle, perforating the clavipectoral fascia with Metzenbaum scissors. After gaining access beneath the pectoralis major muscle, the fibers are carefully separated from the pectoralis minor muscle, ensuring the pectoralis minor remains attached to the chest wall. A large dissector is inserted caudomedially under the pectoralis major to elevate the muscle gently, avoiding contact with the ribs to minimize postoperative pain due to periosteal trauma. The dissection continues until reaching the lower border of the pectoralis major muscle to define the inferior boundary of the pocket, with the anterior axillary line serving as the lateral limit of dissection. (**See Video 2 [online]**, which demonstrates muscle blunt dissection. A large dissector [such as the Solz by Medicon] is inserted caudomedially under the pectoralis major to elevate the muscle gently, avoiding contact with the ribs to minimize postoperative pain due to periosteal trauma. The dissection continues until reaching the lower border of the muscle, creating the lower boundary of the pocket.)

**Video 2.** This video demonstrates muscle blunt dissection. A large dissector (such as the Solz by Medicon) is inserted caudomedially under the pectoralis major to elevate the muscle gently, avoiding contact with the ribs to minimize postoperative pain due to periosteal trauma. The dissection continues until reaching the lower border of the muscle, creating the lower boundary of the pocket.

In the endoscopic variation of the procedure, the dissector is removed and replaced with an endoscopic hook (RZ-Medizintechnik 260-130-016 and 260-137-010 with a 10-mm 30-degree angled optic). The hook is advanced in a caudomedial direction under the pectoralis major muscle until its light becomes visible through the skin at the inferomedial edge of the planned pocket. A small stab incision (2 to 3 mm) is then made at the lateral edge of the IMF, allowing insertion of a monopolar electrocautery instrument into the pocket. Under direct videoendoscopic visualization, the pectoralis major is separated from its medial and inferior attachments to the chest wall, thus creating a dual-plane pocket. Although this auxiliary incision is not strictly necessary, as in most videoscopic procedures (eg, laparoscopy, arthroscopy, robotic surgery), the use of more than one access point facilitates safe and ergonomic dissection.

Close attention must be paid to ensure that the endoscopic hook is advanced symmetrically on both sides. Visualization of the endoscope light through the skin during dissection can assist the surgeon in confirming correct placement and avoiding asymmetry, particularly during the early stages of the learning curve. (**See Video 3 [online]**, which demonstrates dual-plane pocket creation with the semiendoscopic video-assisted technique. An endoscopic hook [eg, the 10-mm breast optic retractor by RZ-Medizintechnik] is positioned through the skin incision, replacing the blunt dissector. The hook is advanced caudomedially under the muscle until its light is visible at the inferomedial edge of the pocket through the skin. A small stab incision is made at the outer edge of the IMF, allowing insertion of a monopolar electrocautery device into the pocket. Under videoendoscopic guidance, the pectoralis major is separated from its medial and lower attachments to the chest wall, creating a dual-plane pocket.) Once the pocket is complete, it is thoroughly inspected for bleeding, and the extent of dissection is reassessed. The implant is inserted with the use of a Keller funnel. (**See Video 4 [online]**, which demonstrates the implant being inserted with the use of a Keller funnel.) The fascia is closed with absorbable polydioxanone 2.0 sutures, and the skin is closed using continuous intradermal Monocryl 4.0 sutures. (**See Video 5 [online]**, which demonstrates the fascia being closed with absorbable polydioxanone 2.0 sutures and the skin with continuous intradermal Monocryl 4.0 sutures.)

**Video 3.** This video demonstrates dual-plane pocket creation with the semiendoscopic video-assisted technique. An endoscopic hook (eg, the 10-mm breast optic retractor by RZ-Medizintechnik) is positioned through the skin incision, replacing the blunt dissector. The hook is advanced caudomedially under the muscle until its light is visible at the inferomedial edge of the pocket through the skin. A small stab incision is made at the outer edge of the IMF, allowing insertion of a monopolar electrocautery device into the pocket. Under videoendoscopic guidance, the pectoralis major is separated from its medial and lower attachments to the chest wall, creating a dual-plane pocket.

**Video 4.** This video demonstrates the implant being inserted with the use of a Keller funnel.

**Video 5.** This video demonstrates the fascia being closed with absorbable polydioxanone 2.0 sutures and the skin with continuous intradermal Monocryl 4.0 sutures.

### Follow-Up

Follow-up evaluations were performed immediately after surgery and daily during the hospital stay. Patients returned for follow-up visits at 1, 2, 4, and 8 weeks and 6, 12, and 24 months postoperatively. Clinical and ultrasound assessments were conducted to detect early and late complications.

We recommend that patients wear an underwire bra with elastic cups for 6 months to help shape the IMF and stabilize the implant position on the chest. We also recommend using a breast band for 2 to 8 weeks, until the pectoralis major muscle has stretched, to prevent implant migration upward and laterally.

In cases of breast induration, undesirable changes in shape, or unexplained asymmetry occurring after the 8th postoperative week, a 3-month course of montelukast 4 mg daily was initiated as a preventive measure against capsular contracture. At each follow-up visit, patients were asked to press their hands together to induce contraction of the pectoralis major muscles. In cases of even minimal distortion, 100 units of botulinum toxin were injected into the inferomedial and superolateral fibers of the pectoralis major to prevent the development of animation deformity. Patients were reevaluated after 6 weeks, and if the distortion persisted, the injection was repeated using the same technique.

### Outcomes

The outcomes evaluated included postoperative complications, such as double-bubble deformity, breast animation deformity (BAD), implant malposition (bottoming out of 0.5 cm or greater), capsular contracture, hematoma, seroma, infection, and implant exposure. Patient satisfaction was evaluated using electronic BREAST-Q modules preoperatively and at 6 and 12 months after surgery. Operative time was measured from the initial skin incision to final skin closure. BAD was evaluated during physical examination according to the classification proposed by Dyrberg et al.,^15^ with grades ranging from 0 (no visible deformity) to 3 (severe deformity visible even at rest or with minimal contraction).

### Statistical Analysis

The primary outcome was the postoperative complication rate. A sample size of 59 patients per group (traditional transaxillary blind versus semiendoscopic video-assisted transaxillary) was calculated to achieve 80% power to detect a 4.5% absolute difference in complication rates, assuming a reduction from 10% under the null hypothesis to 2.5% under the alternative hypothesis, based on previous clinical data. According to data from previous clinical studies, the incidence of capsular contracture in the semiendoscopic video-assisted transaxillary group was estimated to be 0.10. A 2-sided Z test with pooled variance was used. The significance level was set at 0.05.

Continuous variables are reported as mean ± SD and were compared with the Mann-Whitney *U* test. Categorical variables are reported as percentages and compared with the Fisher exact test. All analyses were performed using IBM SPSS version 22.

Patients were analyzed according to intention-to-treat principles. Data are reported and presented in accordance with the updated Consolidated Standards of Reporting Trials criteria.

## RESULTS

**Fig. 1.**
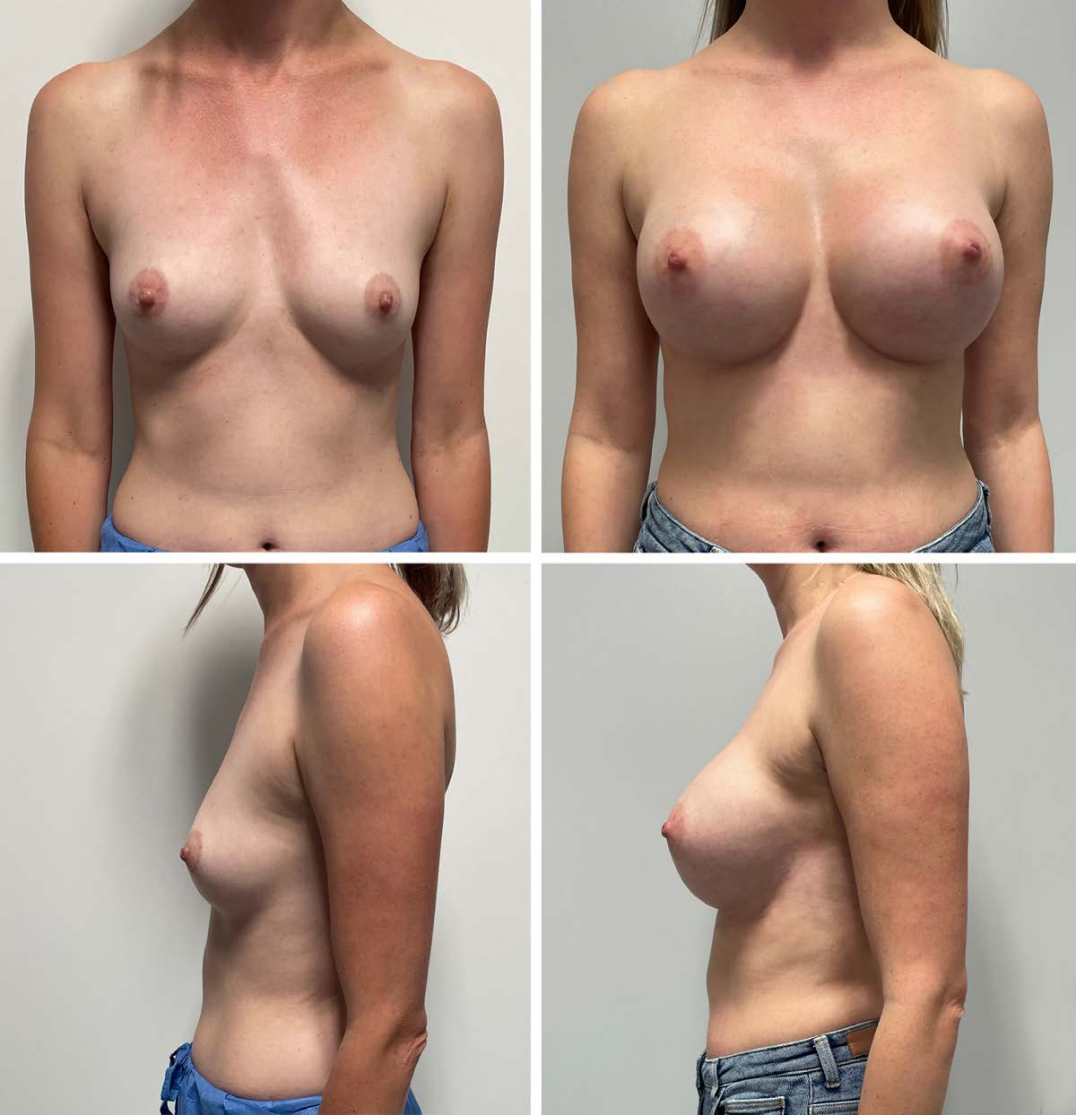
(*Left*) Preoperative frontal and left lateral views of a 30-year-old woman who underwent a transaxillary semiendoscopic dual-plane breast augmentation (355-cc silicone implants). (*Right*) Postoperative views at 2-year follow-up.

**Fig. 2.**
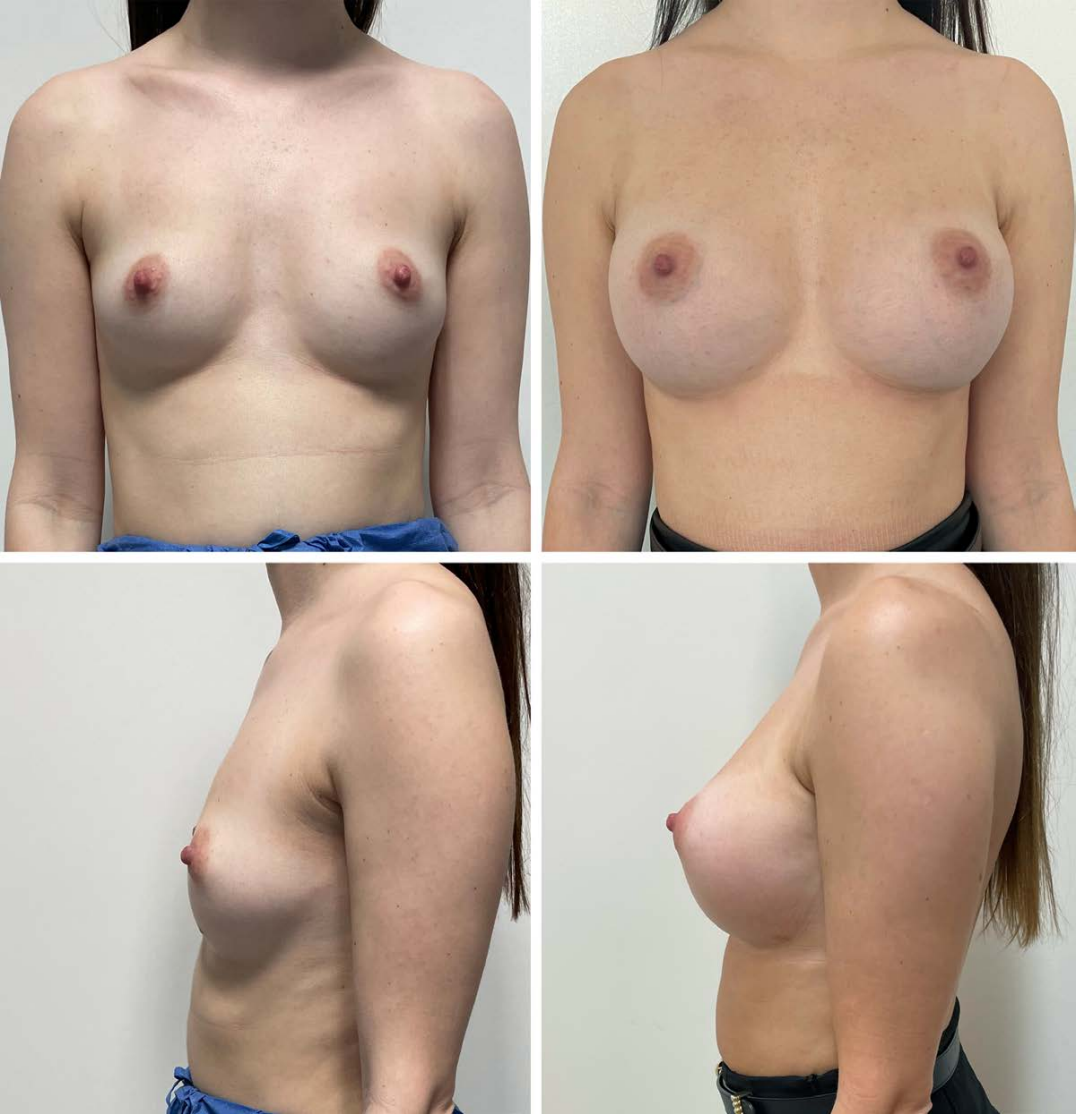
(*Left*) Preoperative frontal and left lateral views of a 31-year-old woman who underwent a transaxillary semiendoscopic dual-plane breast augmentation (360-cc silicone implants). (*Right*) Postoperative views at 2-year follow-up.

From April of 2019 to December of 2022, a total of 118 patients were randomized to the traditional transaxillary blind approach or the semiendoscopic video-assisted transaxillary approach (Figs. 1 and 2). (**See Figure, Supplemental Digital Content 1**, which shows [*above*] the preoperative frontal and left lateral view of a 29-year-old woman who underwent a transaxillary blind breast augmentation [330-cc silicone implants] and [*below*] the postoperative view at 2-year follow-up, https://links.lww.com/PRS/I539.) Fifty-nine patients were randomized to each group. Their baseline characteristics are shown in Table 1.

The mean operative time was significantly shorter for the blind group, averaging 46 minutes, compared with the video-assisted group, in which the average operative time was 58 minutes (*P* = 0.001). The additional time in the video-assisted group can be attributed to the careful dissection and visualization enabled by the endoscope.

No acute complications, such as hematomas, seromas, acute infections, or implant exposures, were observed in either group. However, a significant difference emerged in terms of long-term postoperative complications, typically associated with implant positioning. Minor double-bubble deformities (approximately 1 cm) were observed in 4 patients in the blind group and 1 patient in the video-assisted group. All cases were managed with an office-based surgical correction under local anesthesia, consisting of releasing the adherence between the skin and the capsule in the lower pole through a small stab incision at the nipple, followed by fat grafting to fill the newly created space. Early signs of BAD (Bracaglia grade 1) were significantly more prevalent in the blind group, with 20 cases observed compared with 8 in the semiendoscopic group (*P* = 0.016). All grade 1 BAD cases were treated and resolved after 1 or 2 botulinum toxin injections. However, 2 cases of BAD grade 2 (both in the blind group) showed progression and required surgical revision with endoscopic capsulectomy or capsulotomy and creation of a true dual-plane pocket (*P* = 0.49).

**Fig. 3.**
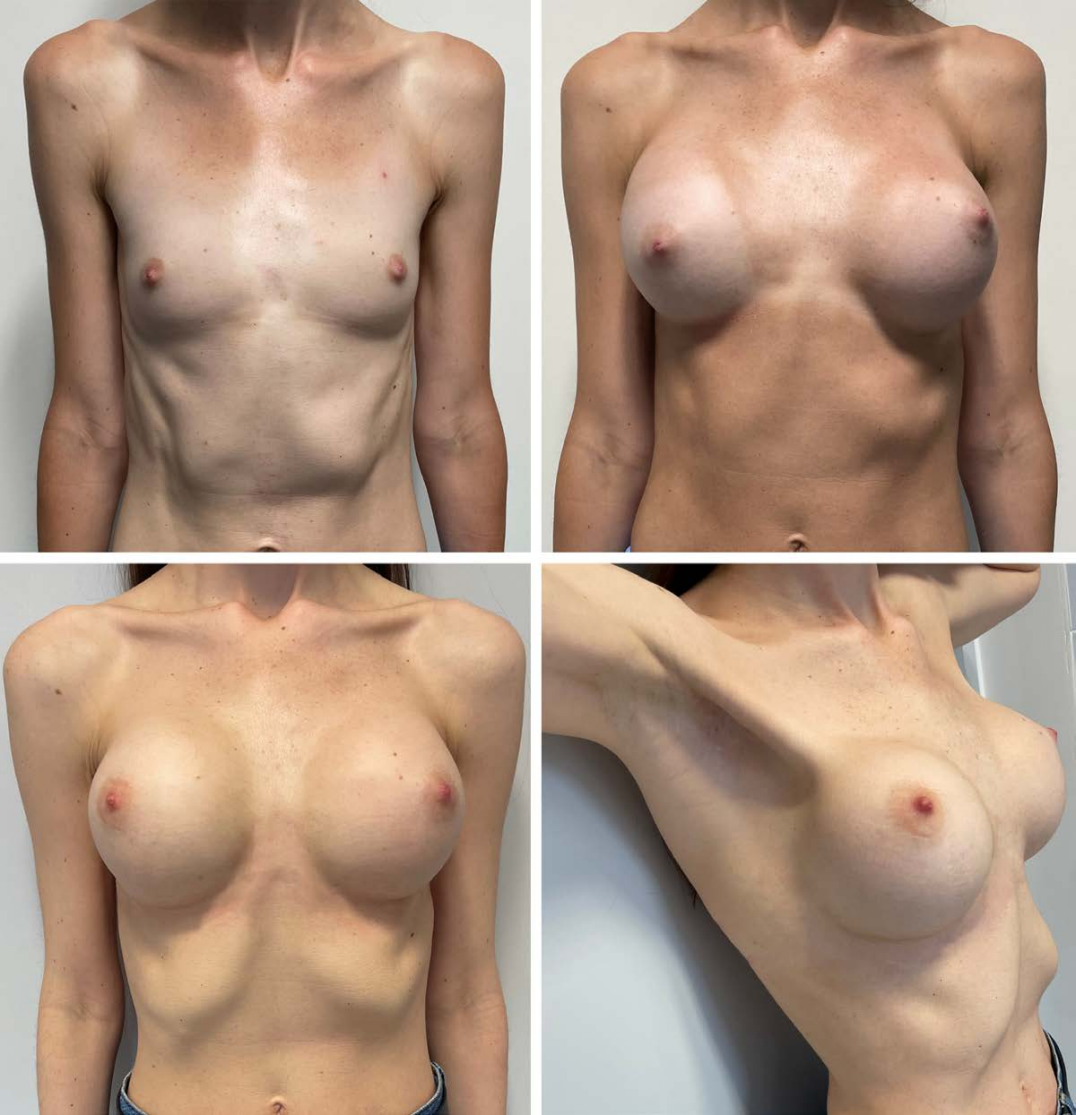
(*Above, left*) Preoperative frontal view of a 36-year-old woman who underwent transaxillary blind breast augmentation (380-cc silicone implants). (*Above, right*) Postoperative view at 1-year follow-up showing a right capsular contracture. (*Below*) Postoperative view at 2-year follow-up after right implant revision surgery with a transaxillary semiendoscopic technique. The scar is barely visible.

Early postoperative symptoms suggestive of capsular contracture (Baker 2) were observed in 3 patients (2 patients in the blind group versus 1 patient in endoscopic group) (*P* = 0.56). Treatment with montelukast and antibiotics was effective in preventing progression in all cases of Baker 2 contracture. Endoscopic capsulectomy was required in 5 cases (4 patients in the blind group versus 1 patient in endoscopic group) of Baker 3 capsular contracture (Fig. 3). No adverse effects were observed after either medical or surgical management of these cases.

Patients in the video-assisted group consistently reported higher satisfaction with the aesthetic results during follow-up visits, which were conducted at 2, 4, and 8 weeks and 6, 12, and 24 months postoperatively (Table 2).

**Table 2.** Operative Outcomes

Outcomes	Blind Transaxillary Approach (*n* = 59)	Endoscope-Assisted Transaxillary Approach (*n* = 59)	*P*
Operative time, min, mean ± SD	46 ± 6	58 ± 5	0.001
Postoperative complications			
Double-bubble deformity	4 (6.8)	1 (1.7)	0.170
Grade 1 BAD	20 (33.9)	8 (3.4)	0.016
Grade 2 BAD	2 (3.4)	0	0.49
Baker 2 capsular contracture	2 (3.4)	1 (1.7)	0.56
Baker 3 capsular contracture	4 (6.8)	1 (1.7)	0.170
Implant malposition	2 (3.38) (1-cm monolateral bottoming out)	1 (1.7) (1-cm monolateral bottoming out)	0.56

Ultrasound assessments at the final follow-up showed stable implant positions in the majority of patients, with minimal evidence of implant malposition in all cases. The meticulous visualization afforded by the endoscope likely contributed to these improved outcomes, allowing surgeons to fine-tune implant placement and minimize the risks of complications such as implant bottoming out and lateralization. Monolateral implant malposition, including minor cases of bottoming out (approximately 1 cm), was observed in 2 patients in the blind group and 1 patient in the video-assisted group (*P* = 0.56). Corrections were successfully performed using minimally invasive office-based procedures, which involved anchoring the IMF skin to the underlying fascia with 2 or 3 sutures of polydioxanone 0 placed through a 0.2-mm stab incision—small enough to allow passage of the needle only. Upon healing, the procedure left no visible scarring, thus preserving the scarless nature of the transaxillary approach.

Both techniques yielded significantly improved postoperative BREAST-Q scores compared with preoperative scores, without significant differences between the 2 techniques. (**See Table, Supplemental Digital Content 2**, which shows preoperative and postoperative BREAST-Q scores, https://links.lww.com/PRS/I540.)

## DISCUSSION

Our findings demonstrate a reduction in long-term postoperative complications when using the semiendoscopic video-assisted transaxillary approach. Baker 3 capsular contracture occurred less frequently in the semiendoscopic group (1 case) compared with the blind group (4 cases). This is likely due to the precise dissection and implant placement afforded by the endoscope, reducing undue trauma to the tissues surrounding the implant, which is a known factor in the development of contracture.^16,17^ In these patients, an endoscopic capsulectomy was performed through the original transaxillary incision. A 10-mm endoscopic retractor and monopolar cautery were used to dissect and excise the fibrotic capsule under direct visualization. The pocket was then irrigated and revised as needed, with replacement of the implant. This minimally invasive approach allowed for complete treatment without compromising the aesthetic result.^13,18,19^

Several preventive measures were implemented to reduce the risk of capsular contracture, inspired by the 13-point plan of Adams et al.^20^ Despite the absence of hematomas in either group, the videoscope allows for the detection and management of minor bleeding, which may contribute to capsular contracture. Moreover, the improved visualization provided by the endoscope allows precise pocket tailoring, minimizing the risk of implant malposition, which can lead to asymmetry and other aesthetic complications.

The reduced incidence of capsular contracture in the video-assisted group supports the hypothesis that minimizing trauma to the surrounding tissues and ensuring proper placement of the implant can prevent the inflammatory processes that lead to contracture. This suggests that the additional time required for the video-assisted technique is a worthwhile investment when considering long-term outcomes and the need for revision surgical procedures.

As noted by Tebbetts,^16^ the blind technique was insufficient for proper separation of muscle fibers from their inferomedial attachments, resulting in higher complication and revision rates in this group.

A dual plane is known to reduce the risk of animation and facilitates filling the lower pole in the ptotic breast. In contrast, the blind approach does not provide visual confirmation of the dissected plane, and it must be assumed that the pocket is entirely submuscular. This likely accounts for the higher prevalence of animation deformity observed in the blind group.

Roxo et al.^12^ compared endoscopic and nonendoscopic transaxillary breast augmentations performed in the subglandular plane. Their study found no significant differences in the positions of the nipple-areola complex or the IMF, or in the incidence of immediate complications between the 2 groups. Notably, by operating in the subglandular plane, they avoided the muscle-related complications identified as primary contributors to adverse outcomes in our study. This aligns with previous reports by Niechajev^21^ and Küntscher^22^ highlighting muscle animation deformities as a significant issue in blind submuscular techniques, often requiring surgical revision.

Aygit et al.,^7^ Roxo et al.,^12^ Price et al.,^13^ Serra-Renom et al.,^8^ and Tebbetts^16^ described fully endoscopic transaxillary techniques and consistently reported complication rates comparable to those observed in our semiendoscopic group, regardless of the dissection plane. However, a notable advantage of our semiendoscopic technique lies in its shorter operative time (58 minutes) compared with fully endoscopic approaches (from 75 to 137 minutes),^7,8,12,13,16^ without compromising outcomes.

Despite its many advantages, the video-assisted transaxillary technique also has some drawbacks. The most notable disadvantage is the increased operative time. Our study showed that surgical procedures in the video-assisted group took an average of 12 minutes longer than those in the blind group. This may not seem significant, but it translates to higher overall operating costs and prolonged anesthesia exposure, which can increase the risk of anesthetic-related complications. Nevertheless, the average operating time in the semiendoscopic group is still far shorter than durations reported for fully endoscopic surgical procedures in the literature.^7,8,13,16^

In addition, the need for specialized equipment adds to the cost and complexity of the procedure. Surgeons require specialized training to perform the video-assisted technique safely and effectively. This may limit the availability of the procedure in certain settings, particularly in smaller clinics or regions where endoscopic equipment is not readily available.

The traditional blind technique, although less precise, has its own advantages. Chief among these is the shorter operative time, as demonstrated in our study. Surgeons who are experienced in the blind technique can complete the procedure more quickly, which can reduce costs and the risks associated with prolonged anesthesia.

The blind technique also requires surgical expertise, as improper execution may increase the risk of complications, as highlighted by Hidalgo.^3^ The overall complication rate is low, but adequate surgical experience remains essential. Hidalgo^3^ emphasized that the success of the blind technique is closely linked to the surgeon’s experience and understanding of anatomical landmarks.

The technical challenges inherent to blind dissection, including the inability to visually confirm the separation of muscular fibers and the creation of a precise implant pocket, underline the potential limitations of this method for less-experienced operators. The semiendoscopic approach may therefore serve as a valuable alternative, particularly in centers where maintaining low complication rates and ensuring reproducibility of outcomes across different levels of surgical expertise is a priority.

Another important consideration is the potential need for revision surgery. Transaxillary revision procedures involving implant replacement and capsulectomy require the use of an endoscope.^13,17,18^ Therefore, to preserve the scarless nature of this approach, the availability of a videoscope and access to the proper equipment must be ensured.

## CONCLUSIONS

The findings of our study demonstrate that the semiendoscopic video-assisted transaxillary approach may offer advantages over the traditional blind method in breast augmentation surgery. Surgeons should consider the semiendoscopic video-assisted method to minimize the risk of asymmetries and deformities.

The most important limitations of our study are linked to the small sample size and the short follow-up, which do not permit definitive conclusions. Further studies with larger cohorts and longer follow-up periods could provide additional insights into the long-term efficacy and cost-effectiveness of these surgical approaches, helping to refine decision-making in breast augmentation surgery.

## DISCLOSURE

The authors declare that they have no relevant conflicts of interest to disclose.

## Supplementary Material



## References

[1] Hoehler H. Breast augmentation: the axillary approach. Br J Plast Surg. 1973;26:373–376. 4759979 10.1016/s0007-1226(73)90044-1

[2] Visconti G Franceschini G Bianchi A. Transaxillary nipple-sparing mastectomy and direct-to-implant breast reconstruction using a simplified endoscopic approach: indications, cosmetic outcomes and technical refinements. Aesthetic Plast Surg. 2020;44:1466–1475. 32468120 10.1007/s00266-020-01792-1

[3] Hidalgo DA. Breast augmentation: choosing the optimal incision, implant, and pocket plane. Plast Reconstr Surg. 2000;105:2202–2216; discussion 2217. 10839422 10.1097/00006534-200005000-00047

[4] Barnsley GP Sigurdson LJ Barnsley SE. Textured surface breast implants in the prevention of capsular contracture among breast augmentation patients: a meta-analysis of randomized controlled trials. Plast Reconstr Surg. 2006;117:2182–2190. 16772915 10.1097/01.prs.0000218184.47372.d5

[5] Pereira LH Sterodimas A. Transaxillary breast augmentation: a prospective comparison of subglandular, subfascial, and submuscular implant insertion. Aesthetic Plast Surg. 2009;33:752–759. 19597863 10.1007/s00266-009-9389-x

[6] Tebbetts JB. Dual plane breast augmentation: optimizing implant-soft-tissue relationships in a wide range of breast types. Plast Reconstr Surg. 2001;107:1255–1272. 11373572 10.1097/00006534-200104150-00027

[7] Aygit AC Basaran K Mercan ES. Transaxillary totally subfascial breast augmentation with anatomical breast implants: review of 27 cases. Plast Reconstr Surg. 2013;131:1149–1156. 23629095 10.1097/PRS.0b013e3182865d68

[8] Serra-Renom J Garrido MF Yoon T. Augmentation mammaplasty with anatomic soft, cohesive silicone implant using the transaxillary approach at a subfascial level with endoscopic assistance. Plast Reconstr Surg. 2005;116:640–645. 16079703 10.1097/01.prs.0000173558.52280.6e

[9] Munhoz AM Fells K Arruda E. Subfascial transaxillary breast augmentation without endoscopic assistance: technical aspects and outcome. Aesthetic Plast Surg. 2006;30:503–512. 16977363 10.1007/s00266-006-0017-8

[10] Gabriel A Abbott EN Maxwell P Sigalove S Perdikis G. Planes in aesthetic breast surgery: is subfascial a misnomer? Aesthet Surg J Open Forum 2024;6:ojae107. 39687073 10.1093/asjof/ojae107 PMC11647268

[11] Fardo D Sequeira Campos MB Pensler JM. Breast augmentation. In: StatPearls. StatPearls Publishing; 2024. 29489168

[12] Roxo ACW Marques RG De Castro CC Aboudib JH. Utility of video-assisted endoscopy in transaxillary breast augmentation. Aesthet Surg J. 2015;35:265–272. 25805280 10.1093/asj/sju076

[13] Price CI Eaves FF Nahai F Jones G Bostwick J. Endoscopic transaxillary subpectoral breast augmentation. Plast Reconstr Surg. 1994;94:612–619. 7938283 10.1097/00006534-199410000-00007

[14] Garcia EB Graziozi AC de Menezes MV Sallum N Ferreira LM. Breast augmentation with transaxillary approach: the advantages of a Z incision: a 344 case experience. Plast Reconstr Surg. 2010;126:112–113.

[15] Dyrberg DL Bille C Gunnarsson GL. Breast animation deformity. Arch Plast Surg. 2019;46:7–15. 30685936 10.5999/aps.2018.00479 PMC6369057

[16] Tebbetts JB. Axillary endoscopic breast augmentation: processes derived from a 28-year experience to optimize outcomes. Plast Reconstr Surg. 2006;118(Suppl):53S–80S. 17099484 10.1097/01.prs.0000247314.92351.99

[17] Hidalgo DA Weinstein AL. Surgical treatment for capsular contracture: a new paradigm and algorithm. Plast Reconstr Surg. 2020;146:516–525. 32842102 10.1097/PRS.0000000000007079

[18] Basile FV Basile AR. Reoperative transaxillary breast surgery: using the axillary incision to treat augmentation-related complications. Aesthetic Plast Surg. 2012;36:323–330. 21938593 10.1007/s00266-011-9810-0

[19] Liu C Chen Y Xu Y Qu Q Wang Z Fan Y. Transaxillary endoscopic approach to capsular contracture following previous breast augmentation: operative technique and clinical outcome. Aesthetic Plast Surg. 2020;44:28–34. 31667548 10.1007/s00266-019-01525-z

[20] Adams WP Jr Culbertson EJ Deva AK. Macrotextured breast implants with defined steps to minimize bacterial contamination around the device: experience in 42,000 implants. Plast Reconstr Surg. 2017;140:427–431. 28841597 10.1097/PRS.0000000000003575

[21] Niechajev I. Improvements in transaxillary breast augmentation. Aesthetic Plast Surg. 2010;34:322–329. 20174802 10.1007/s00266-009-9437-6

[22] Küntscher MV. [Patient satisfaction after primary transaxillary submuscular breast augmentation] (in German). Handchir Mikrochir Plast Chir. 2012;44:227–233. 22932854 10.1055/s-0032-1321862

