# Supplementary figures and images for: Transaxillary Breast Augmentation: A Randomized Controlled Trial Comparing a New Semiendoscopic Video-Assisted Technique versus the Blind Technique
Source: Plast Reconstr Surg. 2025 Oct 20;158(1):43–52. doi: 10.1097/PRS.0000000000012546 (PMC13290058; doi:10.1097/PRS.0000000000012546)

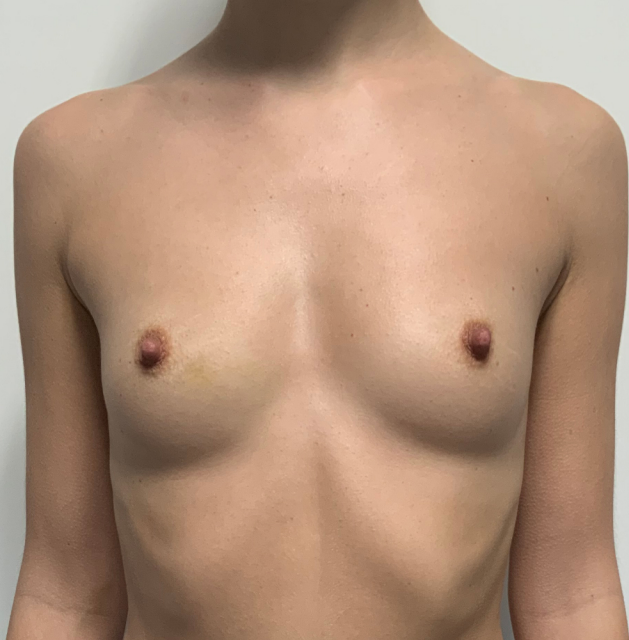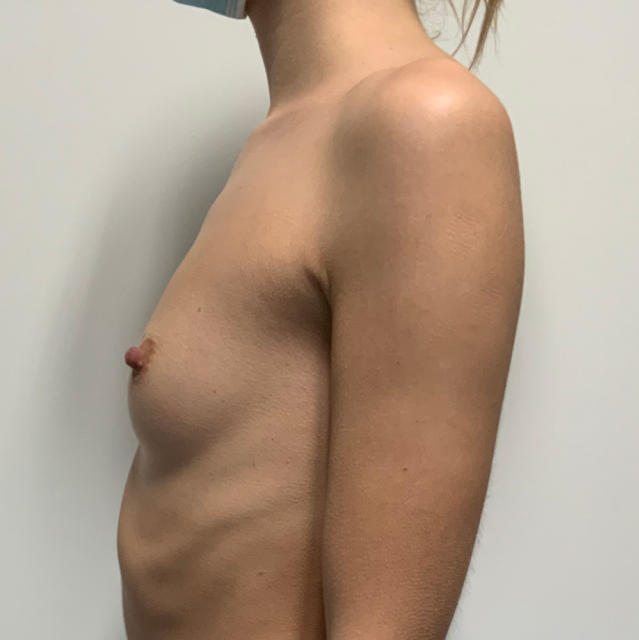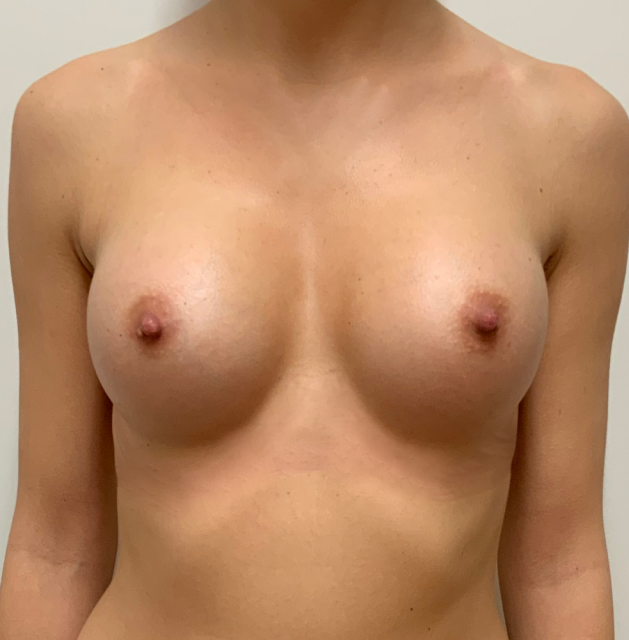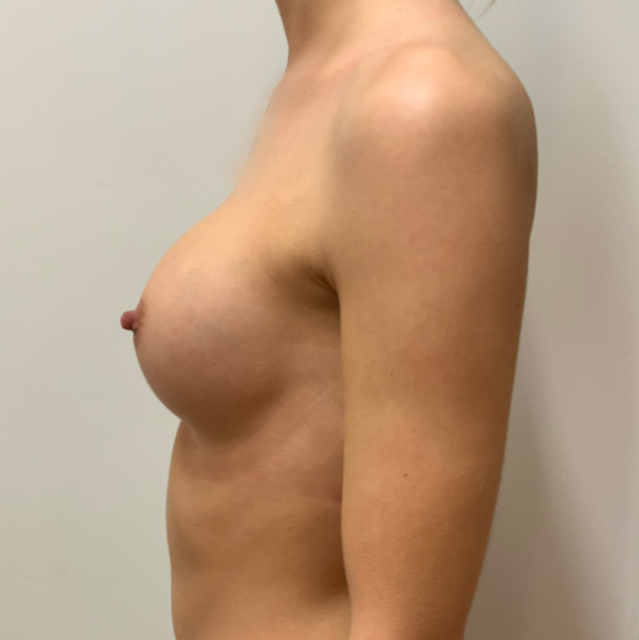

Supplement: Supplementary file 6 [file prs-158-043e-s006.pdf]
