# Supplementary material for: Transaxillary Breast Augmentation: A Randomized Controlled Trial Comparing a New Semiendoscopic Video-Assisted Technique versus the Blind Technique
Source: Plast Reconstr Surg. 2025 Oct 20;158(1):43–52. doi: 10.1097/PRS.0000000000012546 (PMC13290058; doi:10.1097/PRS.0000000000012546)
Supplement: Supplementary file 7 [file prs-158-043e-s007.pdf]

| | Baseline | | | 6 months-FU | | | 12 months-FU | | | Group 1 (p) | | Group 2 (p) | |
|---|---|---|---|---|---|---|---|---|---|---|---|---|---|
| **Category** | **Group 1** | **Group 2** | **P** | **Group 1** | **Group 2** | **P** | **Group 1** | **Group 2** | **P** | **Baseline vs 6 months FU** | **6 vs 12 months FU** | **Baseline vs 6 months-FU** | **6 vs 12 months FU** |
| BREAST-Q **Breast augmentation Module** | | | | | | | | | | | | | |
| **Psychosocial well-being** | 59.2+-8.6 | 58.9+-8.4 | 0.84 | 89.4+-9.2 | 90.1+-8.5 | 0.66 | 88.9+-8.7 | 89.6+-8.8 | 0.65 | 0.001 | 0.76 | 0.001 | 0.75 |
| **Sexual well-being** | 61.1+-9.1 | 60.5+-4.4 | 0.64 | 85.1+-8.1 | 84.9+-8.4 | 0.89 | 84.9+-8.9 | 83.5+-7.8 | 0.36 | 0.001 | 0.89 | 0.001 | 0.35 |
| **Satisfaction with breasts** | 55.1+-4.2 | 54.6+-3.5 | 0.48 | 87.8+-9.1 | 86.9+-8.7 | 0.58 | 88.0+-6.7 | 86.8+-8.7 | 0.4 | 0.001 | 0.89 | 0.001 | 0.95 |
| **Satisfaction with implants** | NA | NA | - | 86.7+-9.1 | 85.9+-9.0 | 0.63 | 86.3+-6.7 | 85.5+-5.9 | 0.49 | 0.001 | 0.78 | 0.001 | 0.77 |
| **Physical well-being** | 56.4+-3.5 | 55.4+-3.4 | 0.11 | 87.1+-8.4 | 86.9+-7.4 | 0.89 | 86.9+-7.6 | 86.5+-6.7 | 0.76 | 0.001 | 0.89 | 0.001 | 0.75 |

**Table, Supplemental Digital Content 2. Pre- and post-operative BREAST-Q score**
